# Reproductive health care for asylum-seeking women - a challenge for health professionals

**DOI:** 10.1186/1471-2458-10-659

**Published:** 2010-11-01

**Authors:** Elisabeth Kurth, Fabienne N Jaeger, Elisabeth Zemp, Sibil Tschudin, Alexander Bischoff

**Affiliations:** 1 Institute of Nursing Science, University of Basel, Basel, Switzerland; 2 Swiss Tropical and Public Health Institute (Swiss TPH), University of Basel, Basel, Switzerland; 3 University Children's Hospital of Basel (UKBB), Basel, Switzerland; 4 University Women's Hospital of Basel, Basel, Switzerland

## Abstract

**Background:**

Dealing with pregnancy, childbirth and the care of newborn babies is a challenge for female asylum seekers and their health care providers. The aim of our study was to identify reproductive health issues in a population of women seeking asylum in Switzerland, and to examine the care they received. The women were insured through a special Health Maintenance Organisation (HMO) and were attending the Women's Clinic of the University Hospital in Basel. We also investigated how the health professionals involved perceived the experience of providing health care for these patients.

**Methods:**

A mixed methods approach combined the analysis of quantitative descriptive data and qualitative data obtained from semi-structured interviews with health care providers and from patients' files. We analysed the records of 80 asylum-seeking patients attending the Women's Clinic insured through an HMO. We conducted semi-structured interviews with 10 care providers from different professional groups. Quantitative data were analysed descriptively. Qualitative data analysis was guided by Grounded Theory.

**Results:**

The principal health problems among the asylum seekers were a high rate of induced abortions (2.5 times higher than in the local population), due to inadequate contraception, and psychosocial stress due to the experience of forced migration and their current difficult life situation. The language barriers were identified as a major difficulty for health professionals in providing care. Health care providers also faced major emotional challenges when taking care of asylum seekers. Additional problems for physicians were that they were often required to act in an official capacity on behalf of the authorities in charge of the asylum process, and they also had to make decisions about controlling expenditure to fulfil the requirements of the HMO. They felt that these decisions sometimes conflicted with their duty towards the patient.

**Conclusion:**

Health policies for asylum seekers need to be designed to assure access to adequate contraception, and to provide psychological care for this vulnerable group of patients. Care for asylum seekers may be emotionally very challenging for health professionals.

## Background

### Female Refugees and Asylum Seekers

Approximately half of the world's international migrant population - 95 million - is female.

Among these migrants, female refugees and asylum seekers are among the most vulnerable. In addition to the hardships of forced migration, they suffer from problems due to their gender [1]. After forced migration, familial protective mechanisms are often no longer adequate, leaving women and girls increasingly at risk of violence and sexual attacks [2,3]. The collapse of social structures further deprives women of resources and support to take care of their children and of their own health [4]. In host countries, female refugees and asylum seekers typically bear the heaviest responsibility for sick family members and children, but at the same time they have less access to information and resources than their male counterparts [5-8].

### Health concerns amongst asylum seekers

Health risk factors for refugees in general include trauma and economic difficulties. In addition, for asylum seekers, there is their uncertain residency status [9-11]. In Switzerland, where our study took place, only approximately 7% of asylum seekers - sometimes after several years of waiting - are finally recognised as refugees and allowed to stay [12]. The fear of expulsion is recognised as a major stressor [13,14].

The most frequently observed health problems among asylum seekers relate to depression and post-traumatic stress [11,15]. In general, asylum seekers are more likely to be diagnosed with psychological problems than the general receiving population [16]. Among physical conditions, gastrointestinal diseases, infections and pain [17,18] are the most frequent disorders. Access to health care is often limited - partly as a result of language barriers [13,19]. Lack of information and difficulties of communication are likely to be especially problematic for asylum seekers, who are often relatively new arrivals.

### Health care for women asylum seekers

Women asylum seekers are also confronted with particular problems related to reproductive health. Many studies have been conducted in order to examine the impact of migration on reproductive health. The outcomes have been varied. A meta-analysis published in 2009 could not show a consistent marker for risk of poor perinatal health outcomes for women with an immigrant background [20]. A study conducted in Canada focusing on post partum health care for mothers and babies did not show a difference in unaddressed concerns of standard postpartum care related to migrant status, when analyses took into account variables which were associated with the migrant status and might have been causing a difference. When these variables were excluded there was a difference in unmet concerns [21].

Studies on perinatal outcomes specifically for asylum seekers show inconsistent results: while a US study of pregnant asylum-seeking women reported increased obstetric complications [22], a British study found no significant differences between asylum-seeking women from Kosovo and a native British control group [23].

However, general assessments of female asylum seekers' or refugees' health are limited, due to there being few instruments which are validated across languages and ethno cultural groups [24].

### The health professionals' perspective

In general, little information is available to physicians, nursing staff and midwives regarding the specific health needs of asylum-seeking women. Physicians in Switzerland report that their training - both basic and postgraduate - has not prepared them adequately for caring for such patients [25,26]. Despite the difficulties health professionals confront when caring for asylum seekers there are very few studies that discuss their problems [13,27].

### Aims of the present study

The aim of the present study was to investigate the reproductive health care provided for women asylum seekers attending the Women's Clinic of the University Hospital in the city of Basel, Switzerland. Our aims were to identify the health needs of asylum seekers attending the Women's Clinic and to investigate the health care they received in a Health maintenance organisation (HMO) specifically established for asylum seekers. In addition, we explored the perceptions of the health care professionals involved, about providing health care for this group in this setting.

## Methods

This study on women's reproductive health was embedded in a larger project examining health and health care provision for all asylum seekers enrolled in the specific HMO model [28,29] of the University Hospital, Basel. The study was approved by the joint ethical committee of the Cantons of Basel Stadt and Basel Land (Ethikkommission beider Basel).

### Study design

We combined quantitative and qualitative methods for data collection and data analysis. The quantitative part was observational, based on records from patients' files and the hospital database and covered the frequencies of diagnoses and medical interventions. The qualitative part included a study of the relevant information from the records in patients' files, and semi-structured interviews with health care providers and textual documents in the patients' files. We analysed the qualitative data guided by the analytic procedures of Grounded Theory [30].

### Study setting

The study took place in the University Women's Hospital, Basel, which is part of the Basel University Hospital. From 2000 through 2003, 979 asylum seekers in the Canton of Basel-Stadt (Basel City) -approximately half of all asylum seekers in the Canton- were assigned to the specific HMO established at the Basel University Hospital. 38% of them were female (n = 375).

This HMO was established specifically for the asylum seeking population. Health Insurance is mandatory for all residents in Switzerland, and people can choose between different insurance models ranging from a free choice in type of care to more restrictive (and generally cheaper) HMO models. Asylum seekers are insured through the Federal Office of Migration who allocates them to an insurance scheme. In response to a drastic increase in numbers of asylum seekers as a result of the war in former Yugoslavia in the 1990s, the Swiss government attempted to reduce health expenditure by limiting the asylum seekers' choice of where to seek care and assigning them to primary health care providers [31].

The University Hospital in Basel, where the present study was carried out, therefore registered as a primary care provider, having established a HMO model specifically for the asylum-seeking population of its area [32]. The University Hospital was paid a capitation fee for each insured asylum seeker. To control expenditures, physicians were instructed to offer 'as much care as necessary' to the patients insured under this HMO model. The definition of 'necessary care' was not clarified but left to the physicians' professional judgement. The Basel HMO model is unique in that it is integrated within the University Hospital, a public institution, and is coordinated by the hospital's Department of Ambulatory Internal Medicine, which provides care for general health issues, and the University Hospital's Women's Clinic, which provides gynaecologic and obstetric care for female asylum seekers.

A specialty of this hospital is its unit of Gynaecological Social Medicine and Psychosomatics (the branch of medical science dedicated to the relation between psychological states, social factors and physical symptoms). Physicians and psychologists working in this unit are trained in psychosomatics. The care offered to patients includes not only joint medical, psychosomatic and psychological care, but also assistance by social workers. The women's hospital also runs an interpreter service for migrant patients.

### Study groups

#### 1. Women asylum seekers

The study group initially consisted of all the 88 asylum-seeking women who received treatment at the University Women's Hospital in- or outpatient clinics from 2000 through 2003. Eight patients had to be excluded from the analysis because their patient charts were missing, leaving a group of 80. Half (50%) originated from former Yugoslavia, the others came from Africa (19%), Asia (16%), Eastern Europe (9%), and other countries (6%). The mean age was 28 years (range: 19-60 years). Only two women were over 45 years, which means that most patients were of childbearing age. The majority of the women (64%) were married at the time of the study. 22% of all the women had never been married. Others were widowed or divorced, or had undocumented family status.

Table 1: characteristics of woman asylum seekers here

**Table 1:** Characteristics of asylum-seeking women in the study group

	N	%
**Age**		
19-20 years	2	2.5%
21-25 years	17	21.5%
26-30 years	28	35%
31-35 years	20	25%
36-40 years	9	11%
>40 years	4	5%
Total	80	100%

**Origin**		
former Yugoslavia		50%
Africa		19%
Asia		16%
Eastern Europe		9%
other countries		6%
Total		100%

Very little information specifically on the asylum-seeking history of the 80 women in our group was accessible to us. An analysis of the total population of asylum seekers enrolled in the hospital's HMO program at this time, concluded that on average, asylum seekers had to wait 1.7 years (maximum 11 years) from the day of filing a request for asylum until the day the decision was made by the authorities [33].

#### 2. Health Care Providers

Our sample of health care providers included members of the different professional groups involved in caring for the asylum seeking patients. There were 3 physicians, 3 members of the nursing/midwifery team, 1 psychologist and 3 interpreters. The people who were invited to participate were those who had been most involved with the asylum-seeking patients insured in the HMO model. They were informed about the study's purpose and signed a statement of informed consent. All the professionals invited agreed to participate.

All the health care professionals were women. One physician and the psychologist were part of the team working at the unit of Gynaecological Social Medicine and Psychosomatics. They had the most intensive contact with the asylum-seeking patients, as these patients were often referred to this unit.

### Data collection

For the quantitative part, we extracted data on asylum-seeking patients from the hospital's electronic database (socio-demographic and insurance data) and from the patients' files (diagnoses and medical interventions). All data were anonymised. For comparative analyses, we extracted data from the University Women's Hospital annual statistics (2000-2003) which documented services provided for the general patient population.

For the qualitative part, we extracted textual data related to health situation and treatment, or to the asylum-seeking process, from patient files - for example, physicians' notes on consultations and referrals, and medical certificates issued for the asylum-seeking authorities. To ensure participants' anonymity specific details (such as nationality, place of residence) were changed or omitted in the extracted text segments.

Information from the health care professionals was obtained in semi-structured interviews, conducted in a quiet room at the hospital or at the university, as chosen by the participants. The interviews were based on an interview guide. Interviewees were encouraged to describe the experience of providing care for asylum-seeking women from both professional and personal points of view. They were asked about their own roles and duties in the HMO-model, about the health concerns of asylum-seeking women as opposed to other women seeking assistance at the University hospital, and about the possibilities and limitations, success and frustration they had experienced when caring for asylum-seeking women. In addition, the physicians were interviewed about their conflicting roles and responsibilities as clinicians, as manager of resources in the HMO, and as writer of certificates for the asylum process. (Only physicians were required to act as resource managers and provide official certificates.) We used probes, like 'What possibilities did you have to help these patients? Which barriers did you experience? What did you consider to be your main duty in caring for the asylum-seeking women?' The interviews were 'audio'-recorded and transcribed verbatim.

### Data analysis

We used descriptive statistical analysis to evaluate quantitative data such as age, frequencies of diagnoses and interventions. Analyses were run using SPSS statistical software.

For the qualitative analysis of transcribed interviews and textual extracts from the patient charts we followed the analytic steps suggested by Grounded Theory methodology. This qualitative approach offers tools to understand human interactions and social processes, taking into account the interdependences of macro-, meso- and micro-levels [30]. To depict social processes comprehensively interview data and different kinds of textual data can be included into the analysis [34]. Data collection and data analysis occurs simultaneously.

We started the process by open coding, which means that we categorized text segments into broad categories or themes (e.g., experiences of violence, offers of professional help, experiencing language barriers, defining one's own responsibility). We continued with axial coding which included examining relationships between categories and connecting them accordingly (e.g., psychosocial stress factors from the past, the present, the future). Finally, selective coding included the organisation of the diverse categories into a framework to explain the phenomenon under study. This framework is depicted and explained in details in the result section.

To strengthen the rigour of the analysis, we discussed the results with experts in women's health, ethics and research. Furthermore, we presented the findings to two participants from the health professionals' group and they judged that the findings mirrored their experiences.

## Results

In part one of this section we review the main reproductive health issues identified among 80 asylum-seeking women and the care they received at the Basel University Women's Hospital. In part two, we detail the range of psychosocial problems encountered in the care of asylum-seeking women, looking at a) stress factors for patients, b) challenges in providing health care from the provider's perspective and c) support strategies for patients that were implemented.

### Part one: Reproductive health issues identified

#### Gynaecological complaints and sexual violence

Information from the files of the 80 patients in the study showed that gynaecological diagnoses most frequently involved urogenital infections (41%), followed by lower-abdominal pain (25%), spontaneous abortions (8%), dysmenorrhoea (5%) and hypermenorrhoea/menorrhagia (5%). Eight files documented that patients had been raped. Of these sexual assaults, six had occurred in the countries of origin, and two in Switzerland.

During the interviews, health professionals and interpreters often mentioned women who had been sexually assaulted in war situations. Any patient who reported such trauma was cared for by a female physician or psychologist from the University Women's Hospital's unit of gynaecological social medicine and psychosomatics.

#### Contraception and undesired pregnancies

Of the 80 patient files, 55 (69%) documented contraceptive measures. The most commonly used contraceptive was the IUD (n = 22, 27% of all patients, 40% of documented uses of contraception), followed by oral ovulation inhibitors (n = 17, 21% of overall patients, 31% of documented contraceptive measures). Overall, 18 unwanted pregnancies were documented. Language barriers were also noted as a reason for women not using contraceptives. The cost of contraceptives, which are not covered by health insurance in Switzerland, was also identified as an obstacle to the use of adequate contraception among women whose resources were very limited, as illustrated in the following file entries:

'The patient comes for fitting of an IUD. But the financing is unclear. Procedure: The financing is being clarified.' (File 16)

'Has no money for the pill.' - [Entry 4 months later:] 'Surgical diagnosis: 24 years old, ninth week of pregnancy, does not want children. Operation: suction curettage, postoperative insertion of an Implanon [hormone releasing rod inserted in upper arm to provide continuous contraception] (File 25).

#### Surgical intervention and induced abortion

The most frequent gynaecological intervention was induced abortion (18 cases), which occurred at a rate far higher than in the general University Women's Hospital population. Data from the 80 files screened yielded an abortion to live-birth ratio of 1:2.5, compared with the overall ratio of 1:7.5 in the University Women's Hospital [35]. Induced abortions were performed surgically (7 cases) or by medication (11 cases). Less frequent was curettage following **s**pontaneous abortion (7 cases) and laparoscopy (4 cases). Other interventions were necessary only in isolated cases. Some types of situations in which asylum-seeking women decided on pregnancy termination are illustrated in the following file entries:

'Patient wants termination of her pregnancy because of severe pain, 'cannot have a child', otherwise no exact reasons can be elucidated, she feels overburdened. History of a miscarriage in 2001, which occurred after a fall on a stairway [after interrogation by the police she was pushed into a dark room and fell down the stairs]. Two days later she miscarried and since then she has felt weak' (File 75).

'No menstruation, unwanted pregnancy, the woman is returning [to the Balkans] to poor living conditions'. - [Three weeks later:] 'Surgical diagnosis: a 27-year-old patient who does not want children, with a psychiatric indication for termination of the pregnancy' (File 59).

Past psychological trauma as well as worries about the future when sent back to the country of origin, were thus identified as some of the reasons for women asylum seekers to ask for induced abortion.

#### Obstetrical issues and perinatal health

The most frequently documented prenatal complications were premature labour (in 15% of pregnancies) and bleeding (11%), followed by gestational diabetes (9%) and retardation of intrauterine growth and anaemia (each 7%).

Two-thirds of the 46 documented deliveries (67%) were spontaneous (see Table 2). Five cases (10%) required an assisted vaginal delivery and 11 (23%) cases an unplanned caesarean section. Planned caesarean sections where not reported. Statistics on mode of delivery were not significantly different from those of the total patient population of the University Women's Hospital.

**Table 2:** Type of delivery: Comparison between asylum-seeking HMO-patients and the total patient population of the Women's Hospital (Years 2000-2003)

	A-Care patients		Total patient population Women's Hospital	
**Type of delivery**				
**Spontaneous**	32	66.7%	4135	64.6%
**Operative vaginal delivery**	5	10.4%	895	14.0%
**Caesarean section**	11	22.9%	1366	21.4%
**Total**	48	100.0%	6396	100.0%

Table 2 (Type of delivery- Comparison) here

Of the 46 babies born, three (6%) were premature (between 35^th ^and 37 ^th ^week of pregnancy). The general rate of premature babies for Switzerland is around 9% [36]. Only one newborn had a birth weight below 2500 grams. On average, they weighed 3470 grams (Standard Deviation 556). One had a malformation of the hand, and two had difficulties in initial neonatal adaption to extra-uterine life.

The primary difficulties listed in the records for the postnatal period were lack of social support and corresponding stress, sometimes leading to exhaustion of mothers. Exhaustion severe enough to require care (counselling, hospitalisation in crisis intervention unit, rest) occurred mainly in mothers in the postnatal period, and in mothers of small children.

### Part two: Psychosocial issues for asylum-seeking women and challenges to the health care professionals

**Figure 1:**
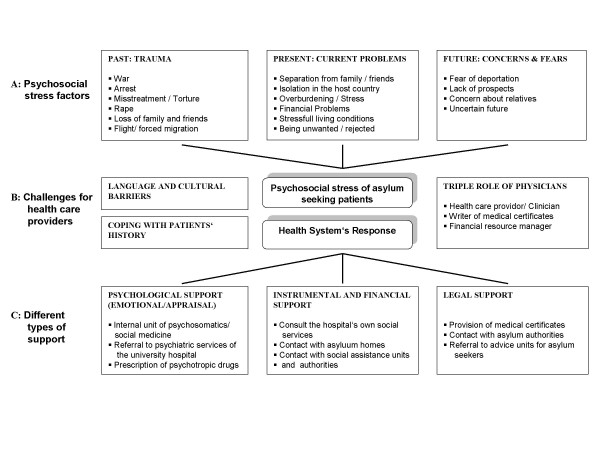
**Dimensions of psychological stress among asylum seekers and the health system's response**.

Health professionals caring for asylum-seeking women were confronted with the psychosocial needs of the patients who came to them for obstetric and gynaecological health care. We grouped the results in the model shown in figure 1, identifying three main areas of interest. We illustrate each of the dimensions with quotations. Area A represents the psychosocial stress factors of asylum-seeking patients, area B the resulting challenges for health care providers in this particular setting, and area C the different types of support (psychological, social and legal, regrouped as health system response) provided for asylum seeking woman by the health system in response to diverse psychosocial needs.

#### Area A: psychosocial stress factors for asylum-seeking patients

Patient files and interview excerpts documented psychosocial stress factors rooted in traumatising past events or in the patients' current life situation, or in fears related to an uncertain future. Rape was mentioned as a factor among many others affecting the women psychologically.

'*Often, if they come directly from a war area they are still very unstable. ... they are traumatised.... And it is not only this rape that they have suffered, but these years of war, with all the alarm and fear, and this leaves enormous marks.' (Interview 5, Psychosomatics Team)*

Often, a combination of stress factors was described:

'Stress factors: Victim of repeated rape, living apart from her husband and two-year-old daughter, imminent deportation to her country of origin. Currently, she is in the third month of pregnancy.... After refusal of her application for asylum, the patient's mental condition has deteriorated further. She appears anxious, restless and tense. She talks of flashbacks, which occur mainly in the evening. She has nightmares and difficulty sleeping. She assures us that she does not feel suicidal.' (File 9)

One health professional interviewed described such an accumulation of stress factors in the patient's history as typical among asylum-seeking patients.

'There were many problems: rapes, being abandoned, and then someone getting shot. It is often complicated.' (Interview 8, Physicians' Team)

#### Area B: Challenges for health care providers

##### Language and cultural barriers

Although the hospital has interpreter services, it was not always possible to find an interpreter for a particular language when needed.

'The language barrier is an enormous problem. It makes it almost impossible even to assess a case history. So (when we have no interpreter available) we look to see whether there is anyone who works in the hospital who knows this particular language. We may find a doctor, for example, who does, and we consult her. But this is not always possible. And then you have to do the best you can and simply get on with the investigations and ultrasound and all that. And somehow you get a picture of this pregnancy and this woman.' (Interview 4, Midwives/Nurses Team)

Whenever possible, patients for whom no interpreters were available were given follow-up appointments with trained interpreters present. But this was not always feasible:

'The people were really nice ... but I just didn't understand them, and I felt that one should understand them, but I simply couldn't. My aim, to do the right thing for a patient, could not be reached. I think that this is what has made me, in the long term, a little hostile when I meet such patients - not actually towards the patients themselves, but simply because I was not able to fulfil my task.' (Interview 9, Physicians' Team)

This illustrates how frustration over communication difficulties can lead to negative feelings linked to the situation being projected on to the migrant patient.

##### Conflicting roles of physicians

Besides linguistic difficulties, physicians faced a range of sometimes conflicting demands that forced them to play a "triple role". In the interviews, they described the potential conflicts between their task of providing patient care, their role in the provision of certificates of health for the asylum authorities, and finally the task of ensuring cost efficacy in the HMO model. As in Switzerland only physicians can issue prescriptions and order exams and treatments, this 'triple role' was particularly a problem for physicians. They were also usually the members of the team required to write official certificates.

Several interviewees stressed their commitment to fulfilling their role as providers of clinical care, irrespective of the patient's residency status. '*I have accepted the task, from the patient or her family, to care for her... I therefore want to do all that I possibly can to ensure that she remains healthy and that things go well for her. That is my duty as a physician.' (Interview 2, Psychosomatics Team)*

Physicians were sometimes required by the authorities in charge of the asylum decisions to assess the state of health of an asylum-seeking patient, along with her prognosis, and write a certificate which could potentially be used in the asylum process. This required a change of perspective: *'I am not being objective when I am caring for a patient. I am on her side as far as the medical aspects are concerned. I just want to help her. And then when at the same time such questions come from the Federal Office for Refugees, something different is expected - in fact an objective opinion. And that is not easy. Somehow one then tries to give consideration to both sides, and that always leads to conflict.' (Interview 2, Psychosomatics Team)*

The third role of physicians is that they are expected to manage financial resources. The basic document of the Basel HMO model for asylum seekers was based on the principle that asylum seekers should receive 'as much [medical care] as necessary'. No list was included of medical services to withhold from HMO patients. It was left to the judgement of each physician to interpret 'as much as necessary' with regard to everyday clinical practice.

*'From the way I see my task, it is in fact more a question of preventing duplications and redundancies, rather than just limiting them ....I would have been a bit concerned - and we have also discussed this - if I had in fact had the feeling that I now have to limit the care being provided. We of course always have to be careful that we do not somehow create a second-class medicine.' (Interview 2, Psychosomatics Team)*.

Other interviewees also describe efforts to treat patients insured under the HMO model according to the same standards as patients with standard insurance coverage:

*'Not that one would have specially reduced any of the investigations. I can't remember that ever happening... In our clinic every woman gets what she needs.' (Interview 8, Physicians' Team*) However, the physicians were aware that treatment of HMO patients '*should cost as little as possible*.' *(Interview 9, Physicians' Team*)

Combining the three roles was demanding for the physicians involved:

*'These requirements differed: on the one hand to be the care giver, to be the patient's advocate in fact, and on the other to act as advocate of the Federal Office for Refugees, and thirdly to be responsible for the organisation, to save costs for the health insurance. But that is simply not possible.' (Interview 9, Physicians' Team) *And: *'A lot was expected of me. [I had to know] all the things that I could do for asylum seekers, with some kind of certificate or something else. And it takes a lot of strength for one to be aware - to make oneself aware - of what one can offer and what one cannot offer. (Interview 2, Psychosomatics Team)*

Working under these conditions was difficult. The problem of conflicting roles meant that beyond their normal commitment to the care of their patients, physicians had to make additional efforts to fulfil the tasks assigned through the government and the HMO model.

##### Coping with the patients' history

Other members of the team caring for asylum seekers did not have to write certificates, but they too had to cope with various problems, including their own response to the patients' often tragic histories. An interpreter said:

Maybe in the beginning it was so difficult because I would always translate for the same patients, who had survived the war. The entire hour they just cried and cried and cried. I remember a woman who had lost her 12-year-old girl during the war. She would say: 'they killed her in front of my house door' and then she cried and cried. Honestly, I would have preferred not to translate for this woman any more. Not because I did not like her but because it was so extremely draining (Interview 1, interpreter).

#### Area C: Different types of social support (psychological, instrumental/financial and legal)

A certain amount of general emotional support was given by members of the health care team. In addition, we identified three types of more specific support provided mainly through specific teams.

##### Psychological support

The most frequent measure for providing psychological care was referral to the University Women's Hospital's unit of Gynaecological Social Medicine and Psychosomatics. Fifty-three percent of participants needed some form of psychological care, which was provided on an outpatient basis by health professionals trained in psychosomatics. Patients received continuous care, supportive counselling and support in overcoming specific difficulties.

'Earlier dreams were discussed. Has lost her family in [...]; she has still not come to terms with this. She has always been afraid. Now she is very exhausted. She tries to dispel the present images and to concentrate on something else' (File 25).

To alleviate the symptoms of mental illness, psychotropic drugs were sometimes prescribed. The health professionals interviewed cited the successful collaboration between the psychosomatics team and the interpreters, along with extended consultations for psychosomatic patients, as favourable factors to provide adequate care for asylum-seeking women.

Emotional support was also provided by members of staff outside the psychosomatics team to asylum seekers:

She was in serious pain. When she saw me, she grabbed my hand and wouldn't let go. She said: Please don't go away, please don't go away' and cried. Because she was so happy to see me, I waited with her until the examination was over. I stroked her hair and said: "be calm, it will be all right". They did not find anything serious. The pain was due to a missed abortion. (Interview 10, interpreter)

##### Instrumental and financial support

Asylum-seeking patients had a wide variety of problems beyond those that had led them to come to the clinic. The care providers consulted the hospital's Social Services department or contacted various authorities or organisations themselves. This support included improving housing conditions, financing contraception and special diets, creating a support network, and bringing families together.

*'The partner lives in [another region] and at present he is not permitted to live with Ms. [...]. It would be extremely helpful for the patient if her partner could live with her and support her at least during the pregnancy and for the first two to three months after the birth' (from a letter to the Official Residents' Services, File 50)*.

##### Legal support in the asylum process

As part of the asylum process, the Federal Office for Migration required treating physicians to provide medical certificates concerning the asylum seekers' health. The physician had to judge the patient's ability to travel, and possible prognoses with or without continued treatment in case of a deportation to the country of origin. As an illustration, here is an extract from a medical certificate provided for the asylum authorities:

Diagnosis: Pregnancy at risk. . Prognosis without treatment: Danger of miscarriage. Prognosis with treatment: Reduction of the danger of miscarriage. Ability to travel from the medical point of view: No. With a pregnancy at risk, travelling would be dangerous because of the stress due to the change of location (File 73).

## Discussion

The principal health problems of the group of asylum seekers attending the Women's Hospital during our study were the high rate of induced abortions, and psychosocial stress symptoms. The latter were due to the experience of forced migration, the women's precarious situation in the host country, and their fears of an uncertain future. Up to 53% of patients required special care from a team specializing in psychosomatics.

Staff found caring for asylum seekers emotionally challenging, and for the physicians there was the additional problem that they had to take on additional roles - writing certificates required by the asylum authorities, and allocating limited resources under an HMO model. They often felt that these activities were in conflict with their desire to provide support and the best possible care for this extremely disadvantaged group of patients.

### Contraception and induced abortion

Despite the high level of medical care available in Switzerland not all the women had full access to adequate contraception. We found that there were problems owing to difficulties of understanding, and also financial hurdles. It is likely that in some cases the negative attitude towards contraception which was identified in a Danish study [37] as a risk factor for abortion in migrant women might have also been of importance, but this could not be identified in our retrospective study. However, we found a link in some cases between limited resources, making contraception inaccessible even for women who wanted it, and the lack of contraception resulting in unwanted pregnancies and abortion.

Analysis of our data showed an abortion rate per conception 2.5 times higher than that of the local population. A Dutch study [38] shows a similar trend with a 1.6 times higher abortion rate in asylum-seeking women than in the entire population. They also showed a decrease in abortion rates with the length of stay in the Netherlands. Our data did not allow us to analyse how many of the pregnancies were conceived before the mother arrived in Switzerland, or whether the rate of abortions would have decreased with the length of stay in the host country.

### Psychosocial needs

More than half of the women in our study group required special care from the psychosomatic team of the women's clinic, which emphasises how much they needed help in coping with many aspects of their lives. The trauma many of them had suffered in their homelands, the experience of forced migration, the difficult present situation in the host country, and uncertainty about the future, were the main psychosocial stressors identified.

High rates of sexual assault before and after arriving in the host country are likely to have increased the need for support.

The analysis of files indicated that the high rate of induced abortions was also connected to the women's socio-economic situation, in which some asylum-seeking women felt ill-equipped to care for a newborn child. For mothers of newborns and small children, various stress factors, including the lack of a familiar social environment, left them prone to exhaustion. Our results are supported by the study carried out by Zahorka and Blöchliger [39] who, in an analysis of the reproductive health needs of female immigrants in Switzerland, identified a lack of social support as the main problem among mothers of infants. Most asylum seekers have fewer social and family contacts than other migrants [40] and are thus even more likely to be socially isolated.

### Perspectives of health care providers

In this study we also asked health care providers about their experience of caring for asylum-seeking women, and whether they encountered any special problems. As in earlier studies on health care for migrants, health professionals described language barriers and the legal/social difficulties faced by asylum-seeking patients as challenges they were only partly able to meet [26,41]. We also found that many health professionals considered that caring for this population if often more laborious and emotionally challenging than with other patients, not only because of communication difficulties but also because of the demands for information and documentation made by the authorities in charge of asylum decisions, and the need to comply with the requirements of the HMO. The emotional challenges sometimes even resulted in the projection of negative feelings on to the asylum seekers. As was also found in study in Geneva [42], the emotional challenges were particularly difficult for the interpreters, who had to translate asylum seekers' traumatic experiences. Sharing the patient's background and maybe even similar experiences can make interpreting even more painful for them [42].

Analysis of the interviews with physicians confirms that for them, meeting their obligations towards their patients, while fulfilling the official requirement of issuing neutral and objective health certificates, was a cause of conflict. The possibility that physicians might support patients to the extent that they write deceptive certificates to improve patients' chances in the asylum process has been discussed in the literature [43]. There is no suggestion in our data that this was happening in the Basel clinic.

Another stress factor for physicians was that the HMO model that provided insurance for the asylum seekers required that they should limit health care measures for these patients to doing 'as much as necessary'. In complying with this demand, health professionals felt they ran the risk of compromising their professional/ethical obligation to treat all patients equally, irrespective of their status or country of origin [44]. In fact, in the public women's hospital in which our study took place, no explicit rationing of care provided for asylum-seeking patients could be identified. We did note that no elective caesarean sections had been offered to our relatively small sample of mothers, but no significant difference in the overall mode of delivery compared to the local population was observed during the 3-year period of our study.

### Strengths and limitations

The strength of this study, which combined quantitative and qualitative approaches, lies in the cross-validation and better interpretability of results (e.g., lack of finance possibly contributing to high abortion rates). A further strength was that the study population of asylum-seeking women comprised all patients getting gynaecologic and obstetric care who were insured in an HMO model which covered half of the asylum-seeking population of Basel, which is one of the largest Swiss cities. This sample can therefore be considered quite representative of the population of women asylum seekers in Switzerland during the time period of the study. The HMO setting did not allow medical consultations outside the study setting, so this potential confounding factor was absent.

However, the results of this study have to be interpreted in the light of certain limitations. Some of these apply to the data on the distribution of health conditions. Retrospective analysis of patients' documentation may result in incomplete information. Small numbers and a short period of investigation did not allow analysis of rare conditions or changes over time, nor an in-depth analysis of pregnancy outcomes. Comparing findings with a standard group was limited to comparing with basic general hospital statistics available.

A further limitation was that the method of collecting data about patients also did not give any information on their perceptions of their health needs and the care they received.

Concerning the perspectives of health professionals, given the small numbers which were interviewed in each professional group, the findings may not reflect the entire range of professionals' experiences when providing care for asylum seekers. In addition, this study was carried out in a hospital setting where highly developed services provided by interpreters, social workers and psychosomatic specialists support care provision for asylum-seeking patients. In other settings, where such services are less developed, providing care for this group may be an even greater challenge for health professionals.

### Implications for practice and future research

Providing adequate care for asylum-seeking women is a challenging task for health care providers. Language barriers can be largely overcome by having the services of well-trained professional interpreters who should be available when needed. This is crucial both for the patients' sake and to avoid frustration in health care providers.

Additional training and ongoing support for health care providers may also be necessary, especially when no health professionals specialised in the care of asylum seekers, in psychosomatics or in intercultural issues are available in an institution. Training and support are needed not only because of the emotional challenges resulting from the situation, but because the patients do not only need medical care, but very often suffer from severe psychosocial problems arising from the stressful situation they are in. They may have a wide variety of problems, ranging from financial limitations and the problems of coping with life in a new culture, to the stress resulting form the asylum process, and the traumatic experiences they underwent before they arrived in the host country.

There was some evidence in this study that financial difficulties could prevent women from practising adequate contraception in a setting like Switzerland, where contraceptives have to be paid for and are not included in insurance schemes. This can potentially result in unwanted pregnancies followed by induced abortion. There is a good case for making contraception free of charge.

Further studies of reproductive health in asylum-seeking women with a larger cohort would bring further insights into their needs, and help to optimise care and preventive measures such as contraception.

An additional problem is that if the State system also charges health professionals treating asylum seekers with tasks unrelated to therapy, this may compromise the relationship between them and their patients. A solution would be the separation of therapeutic and non-therapeutic roles.

Attention should also be paid to stressors that could potentially affect health professionals and their work: the need for support and training of health care providers caring for vulnerable populations should be investigated further. Special attention should be given to the needs of interpreters who may - through potentially sharing the same backgrounds and similar experiences as the patients - be emotionally particularly challenged. The effect on health care providers of working in a restrictive HMO setting, where they do not only have to carry out their traditional clinical tasks but must also cope with increasing managerial responsibilities and financial restrictions, may also warrant further study.

## Conclusion

Even in a country like Switzerland, with a high level of resources, policy makers' and professionals' ongoing efforts are required to ensure appropriate health care provision for asylum-seeking women and to meet their particular needs, especially concerning adequate contraception and psychosocial care. It is necessary to ensure that there is sufficient support for the health professionals involved, since care for asylum seekers may be emotionally very challenging for them.

## Competing interests

The authors declare that they have no competing interests.

## Authors' contributions

Study conception and design: EK, AB, EZ, ST. Coordination and implementation of the study: EK, AB, ST. Data collection: EK. Data management and analysis: EK, AB. Drafting of manuscript: EK. In-depth revision of manuscript, literature research and reevaluation of findings: FNJ. All authors read and approved the final manuscript.

## Pre-publication history

The pre-publication history for this paper can be accessed here:

http://www.biomedcentral.com/1471-2458/10/659/prepub
